# Thin-Slice Prostate MRI Enabled by Deep Learning Image Reconstruction

**DOI:** 10.3390/cancers15030578

**Published:** 2023-01-18

**Authors:** Sebastian Gassenmaier, Verena Warm, Dominik Nickel, Elisabeth Weiland, Judith Herrmann, Haidara Almansour, Daniel Wessling, Saif Afat

**Affiliations:** 1 Department of Diagnostic and Interventional Radiology, Eberhard Karls University Tuebingen, Hoppe-Seyler-Strasse 3, 72076 Tuebingen, Germany; 2 Institute for Pathology and Neuropathology, University Hospital of Tuebingen, Eberhard Karls University Tuebingen, 72076 Tuebingen, Germany; 3 MR Applications Predevelopment, Siemens Healthcare GmbH, Allee am Roethelheimpark 2, 91052 Erlangen, Germany

**Keywords:** MRI, deep learning, prostate, thin-slice, image reconstruction

## Abstract

**Simple Summary:**

Multi-parametric MRI (mpMRI) of the prostate is one emerging tool for early detection of clinically-significant cancer. Standard acquisition protocols provide a slice thickness of 3 mm in T2-weighted TSE imaging. However, thin-slice imaging might be superior for the assessment of the prostate parenchyma. The main disadvantage of thin-slice imaging is related to prolongation of acquisition time. In this study, similar acquisition times could be enabled by deep learning image reconstruction of thin-slice imaging as compared to standard 3 mm T2 imaging. The results demonstrate superior image quality and diagnostic confidence in thin-slice imaging.

**Abstract:**

Objectives: Thin-slice prostate MRI might be beneficial for prostate cancer diagnostics. However, prolongation of acquisition time is a major drawback of thin-slice imaging. Therefore, the purpose of this study was to investigate the impact of a thin-slice deep learning accelerated T2-weighted (w) TSE imaging sequence (T2_DLR_) of the prostate as compared to conventional T2w TSE imaging (T2_S_). Materials and Methods: Thirty patients were included in this prospective study at one university center after obtaining written informed consent. T2_S_ (3 mm slice thickness) was acquired first in three orthogonal planes followed by thin-slice T2_DLR_ (2 mm slice thickness) in axial plane. Acquisition time of axial conventional T2_S_ was 4:12 min compared to 4:37 min for T2_DLR_. Imaging datasets were evaluated by two radiologists using a Likert-scale ranging from 1–4, with 4 being the best regarding the following parameters: sharpness, lesion detectability, artifacts, overall image quality, and diagnostic confidence. Furthermore, preference of T2_S_ versus T2_DLR_ was evaluated. Results: The mean patient age was 68 ± 8 years. Sharpness of images and lesion detectability were rated better in T2_DLR_ with a median of 4 versus a median of 3 in T2_S_ (*p* < 0.001 for both readers). Image noise was evaluated to be significantly worse in T2_DLR_ as compared to T2_S_ (*p* < 0.001 and *p* = 0.021, respectively). Overall image quality was also evaluated to be superior in T2_DLR_ versus T2_S_ with a median of 4 versus 3 (*p* < 0.001 for both readers). Both readers chose T2_DLR_ in 29 cases as their preference. Conclusions: Thin-slice T2_DLR_ of the prostate provides a significant improvement of image quality without significant prolongation of acquisition time.

## 1. Introduction

Prostate cancer is one of the most common cancer diseases in men [1]. However, overdiagnosis and overtreatment of prostate cancer due to detection of clinically non-significant cancers should be avoided. Multiparametric magnetic resonance imaging (mpMRI) of the prostate represents one of the emerging techniques for gentle and accurate non-invasive detection of clinically significant prostate cancer [2,3]. T2-weighted turbo spin echo (TSE) imaging is of utmost importance for sufficient organ evaluation. To achieve high image quality, the prostate imaging and reporting data system (PI-RADS) was published with recommendations regarding the acquisition protocol [4]. Usually, a slice thickness of 3 mm is applied for a compromise between sufficient accuracy and acceptable examination times [5]. However, there might be a chance of missing small cancers via this setting. Furthermore, judgement of capsule infiltration can be challenging using standard imaging. Therapeutic management is important; therefore, imaging should be as accurate as possible. Unfortunately, due to the already lengthy mpMRI prostate protocol, thin-slice T2-weighted TSE imaging is not compatible for daily routine clinical imaging using standard reconstruction pathways. However, with the increasing importance of artificial intelligence, a possible solution for this dilemma might be deep learning image reconstruction (DLR), which has been applied with promising results regarding acquisition time reduction and improvement of image quality in T2-weighted imaging of the prostate, as well as in gradient echo (GRE) imaging of the abdomen [6,7,8,9]. These techniques allow undersampling to an extent that was previously not feasible with conventional reconstruction methods, leading to drastic acceleration of MRI examinations. Apart from acceleration, these algorithms can also be used to compensate signal loss due to higher morphological resolution. Promising results of thin-slice imaging, including DLR, have been presented in brain imaging [10]. Therefore, a combination of thin-slice imaging and DLR, including acquisition time reduction to avoid lengthy examination times, might constitute a promising approach to improve diagnostic imaging procedures. 

Therefore, the aim of this study was to investigate the impact of a DLR thin-slice T2-weighted turbo spin echo (TSE) sequence of the prostate on image quality, artifacts, and diagnostic confidence.

## 2. Materials and Methods

### 2.1. Study Design

This monocentric prospective study was approved by the institutional review board. Informed consent was obtained from all participants. Thirty patients who were referred for mpMRI due to suspicion or relapse of prostate carcinoma between September 2021 and April 2022 were included in this study. Exclusion criteria were MRI unconditional implants.

### 2.2. MRI Acquisition Parameters

All examinations were performed using a 3T clinical MRI scanner (MAGNETOM Vida and Vida^fit^, Siemens Healthcare; Erlangen, Germany). The acquisition protocol consisted of standard T2-weighted TSE imaging (slice thickness: 3 mm) in 3 planes (T2_S_) followed by thin-slice (slice thickness: 2 mm) DLR axial T2-weighted TSE imaging (T2_DLR_). Subsequently, the remaining sequences of the mpMRI protocol were acquired (DWI, T1-weighted imaging, and dynamic contrast-enhanced (DCE) imaging). Acquisition time for axial T2_S_ was 4:12 min as compared to 4:37 min for T2_DLR_. Detailed acquisition parameters are displayed in Table 1. For deep learning image reconstruction, an unrolled variational network [11] was used that has been previously explored with a focus on acquisition time reduction [6,7]. The network was trained on more than 10,000 slices obtained from volunteer acquisitions using various clinical 1.5T and 3T scanners (MAGNETOM scanners, Siemens Healthcare, Erlangen, Germany), and integrated into the scanner reconstruction pipeline for prospective use.

### 2.3. Image Analysis

All imaging datasets were evaluated in a blinded random order reading independently by two different radiologists with three and six years of experience in prostate imaging. A dedicated workstation (GE Centricity PACS RA 1000; General Electric Healthcare) was used for study readings. Datasets consisted of axial T2-weighted imaging, diffusion-weighted imaging (DWI) and DCE imaging forming 60 datasets (30 datasets of T2_S_ and 30 datasets of T2_DLR_). As thin-slice imaging was only performed in the axial plane, coronal and sagittal T2_S_ was not available for study readings. All datasets were evaluated regarding the following criteria by both radiologists using a Likert-scale ranging from 1–4, with 4 being the best image impression: extent of noise (1: high level of noise; 2: intermediate level of noise; 3: low level of noise; 4: almost no noise), sharpness (1: severe blurring; 2: intermediate blurring; 3: slight blurring; 4: no blurring), artifacts (1: non-diagnostic due to distortion; 2: intermediate image distortion; 3: slight image distortion; 4: no artifacts), lesion detectability (1: indistinct lesion boundaries; 2: slight blurring of lesion boundaries; 3: good depiction of lesion boundaries; 4: excellent lesion depiction), overall image quality (1: poor image quality; 2: intermediate image quality; 3: good image quality; 4: excellent image quality), and diagnostic confidence (1: non-diagnostic; 2: restricted diagnostic confidence; 3: good diagnostic confidence; 4: excellent diagnostic confidence). Furthermore, suspicion of extraprostatic growth was assessed. PI-RADS and T2 scoring were performed, including lesion size measurement of the most suspicious lesion in cases of PI-RADS ≥ 3. For reading, PI-RADS Version 2.1 was applied. After a time gap of 4 weeks, a second reading was performed regarding the preference of T2_S_ versus T2_DLR_. For this reading session, both image datasets (without identifying information) were shown simultaneously next to each other.

### 2.4. Statistical Evaluation

Proprietary statistical software was used for analysis (SPSS Statistics Version 26; IMB, Armonk, NY, USA, and JMP 16; SAS Institute, Cary, NC, USA). The Wilcoxon signed rank test for paired data was used for comparison of image quality parameters. Inter-reader agreement was calculated using Cohen’s kappa. *p*-values below 0.05 were regarded as significant.

## 3. Results

Thirty patients were enrolled in this study and successfully examined. In one patient, post prostatectomy was revealed after imaging status. The mean patient age was 68 ± 8 years (100% male). The median prostate specific antigen (PSA) level was 7.5 ng/mL with an interquartile range (IQR) of 2.7–12 ng/mL. Twenty patients underwent biopsy, which revealed no malignancy in seven cases. Four patients underwent prostatectomy after the MRI examination. Further details are shown in Table 2.

### 3.1. Image Quality Analysis

Inter-reader agreement ranged from 0.69 for T2_S_ to 0.80 for T2_DLR_. The results from the more experienced reader 1 are displayed in the following section for better readability using median and IQR in parentheses. All results are available within Table 3.

**Figure 1:**
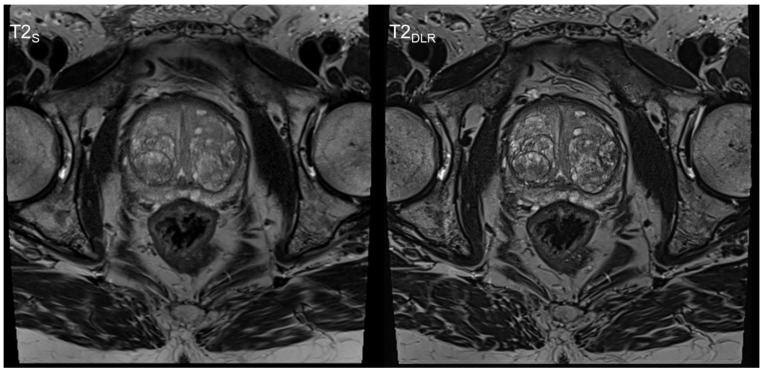
Sixty-year-old man with elevated PSA level. PI-RADS 2 score. No biopsy was performed. Deep learning image reconstructed thin-slice imaging (T2_DLR_) on the right-hand side with superior levels of sharpness and lesion detectability.

Image noise levels were evaluated to be significantly worse in T2_DLR_ as compared to T2_S_, with a median of 3.5 (3–4) versus 4 (4–4) (*p* < 0.001), respectively. However, the sharpness of the images and the extent of artifacts were evaluated to be better in T2_DLR_, with a median of 4 (4–4), versus T2_S_ with a median of 3 (3–3) (both *p* < 0.001) (Figure 1).

**Figure 2:**
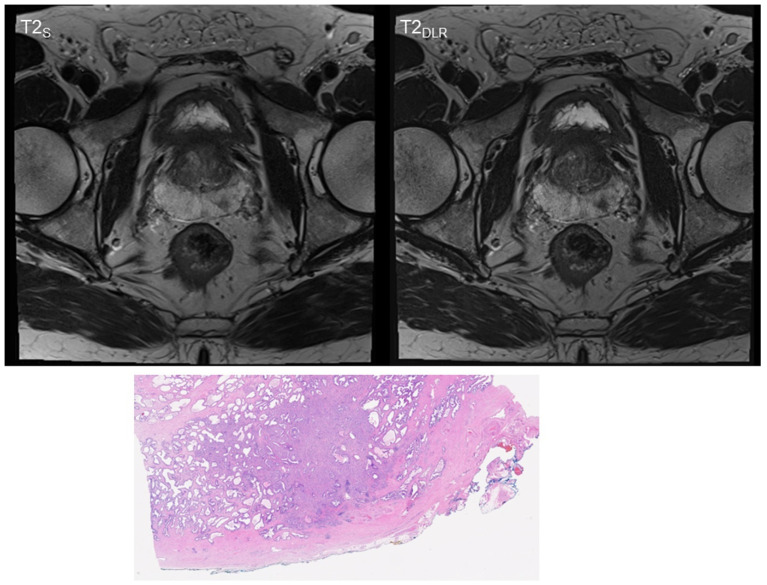
Sixty-three-year-old man with suspicion of prostate cancer. Deep learning image reconstructed thin-slice imaging (T2_DLR_) on the right-hand side with superior levels of sharpness and lesion detectability. PI-RADS 4 lesion in the left posterior peripheral zone. Biopsy was performed and resulted in Gleason 7a prostate carcinoma. The patient underwent prostatectomy that resulted in local T2 stage.

**Figure 3:**
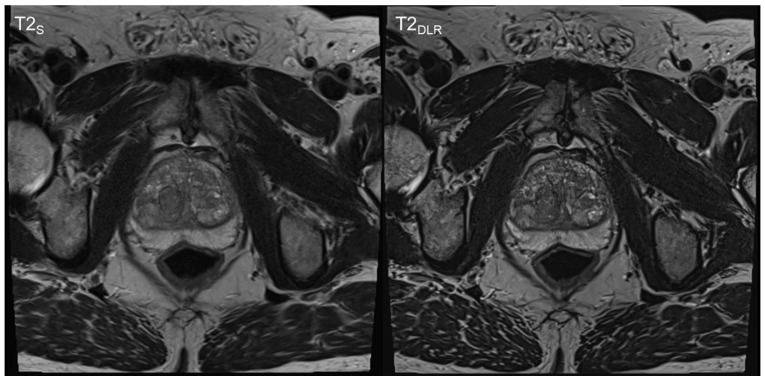
Sixty-nine-year-old man with suspicion of prostate cancer. Deep learning image reconstructed thin-slice imaging (T2_DLR_) is shown on the right-hand side and standard imaging (T2_S_) on the left-hand side. T2_DLR_ shows improved levels of sharpness and improved delineation of the prostate parenchyma. Biopsy revealed no malignancy.

Lesion detectability was also rated to be superior in T2_DLR_, with a median of 4 (4–4) versus a median of 3 (3–4) in T2_S_ (*p* < 0.001). The assessment of overall image quality resulted in superior ratings for T2_DLR_, with a median of 4 (4–4), as compared to a median of 3 (3–3) for T2_S_ (*p* < 0.001). Diagnostic confidence levels were also rated higher for T2_DLR_, with a median of 4 (4–4) in comparison to a median of 3 (3–4) for T2_S_ (*p* < 0.001). For image examples, see Figure 2 and Figure 3.

Both readers chose T2_DLR_ as their preference in 29 cases.

### 3.2. T2 and PI-RADS Scoring and Lesion Size Measurement

In eight cases reader 1 diagnosed a T2 score of 4 (both T2_S_ and T2_DLR_) and in seven cases a T2 score of 5 (both T2_S_ and T2_DLR_). In seven cases reader 2 diagnosed a T2 score of 4 (both T2_S_ and T2_DLR_) and in eight cases a T2 score of 5 (both T2_S_ and T2_DLR_). Reader 1 chose a PI-RADS 4 score in ten cases (both T2_S_ and T2_DLR_), whereas reader 2 chose a PI-RADS 4 score for nine lesions (both T2_S_ and T2_DLR_). No significant difference was found between the sequences or between the readers. Further details are shown in Table 4.

## 4. Discussion

The findings demonstrate that thin-slice T2-weighted TSE imaging, including DLR reconstruction, is feasible without significant prolongation of acquisition time. Furthermore, an improvement in image quality can be achieved via this approach. This study is in line with previous publications investigating the success of deep learning applications for MRI in general, as well as for prostate imaging in particular [6,7,8,9,12,13,14]. It was previously shown that DLR could be applied for acquisition time reduction in prostate mpMRI for T2-weighted imaging as well as for DWI [6,7,15]. Given the economic potential as well as the shortage of scanner capacities, pathways for acquisition time reduction have been of primary interest in most recent DLR studies. However, apart from acquisition time reduction, this study highlights other possible and valuable areas of applications for DLR. Improvement of image quality via acquisition of high-resolution images might lift diagnostic capabilities of prostate MRI to a next level, with an astonishing impression of anatomic details that are as yet unseen. Early detection of suspicious lesions, as well as improved delineation of cancers in staging situations, might display benefits for patient care. However, in this present study no significant difference was found regarding cancer detection. Therefore, further studies in a prospective manner with larger samples sizes are necessary to investigate this issue, including analysis of additional thin-slice sequences (e.g., DWI).

This study demonstrates the various advantages and potentials of DLR in MRI. The benefits of DLR were already presented in GRE imaging of the upper abdomen as well as in TSE musculoskeletal imaging [8,9,13,16,17,18,19]. Furthermore, interesting approaches were analyzed for ultra-fast sequences in the upper abdomen [12,20,21]. In contrast to denoising post-processing procedures only, the additional application of DLR allows a larger extent of undersampling [22,23]. These developments might help alleviate the inherent and lingering shortage of MRI capacities. Additionally, these changes could lead to the implementation of screening programs and routine staging examinations with MRI instead of computed tomography. The latter would be especially helpful for younger patients due to the avoidance of radiation exposure. Furthermore, reduction of acquisition time can help increase patient comfort, which would be of particular interest for elderly and severely ill patients.

However, changes in deep learning technologies on radiological workflows are not limited to image reconstruction tasks. Many studies have been presented regarding diagnostic support tools or automatic detection of certain findings, e.g., in chest, musculoskeletal, or brain imaging [24,25,26]. Furthermore, survival prediction models using radiological image data and deep learning technologies were analyzed with promising results [27]. However, a major concern within the radiological society is related to low levels of trust with these results. On the one hand, this is probably related to missing understanding of certain algorithms, and on the other hand, to the unknown background (“black box” character) of certain processing steps. Furthermore, many radiologists fear loss of importance due to predictions of fully automated image analysis in the future [28,29]. This is why the implementation of DLR might be an “appetizer” for further applications of artificial intelligence techniques in radiology. Almost all radiology departments are striving for workflow optimizations and seeking ways to shorten examination times, increase patient throughput, and simultaneously improve image quality. These aims can all be addressed using modern DLR. Furthermore, the increasing number of studies with striking positive results demonstrate that the fear of image alterations affecting diagnosis (e.g. “vanishing of lesions”) is not necessary and not appropriate anymore with reasonable undersampling. However, it is advisable to avoid extreme undersampling approaches resulting in potentially non-diagnostic image datasets. With growing confidence in these novel technologies, these developments might be the beginning of a new era of radiological imaging.

A major limitation of this present study is its small sample size. Further studies with larger sample sizes are necessary to investigate these issues. This should ideally be performed in patients who undergo prostatectomy after an MRI examination to obtain a complete histopathological work-up that allows investigation of capsule infiltration and lesion detection. Another limitation is the analysis of only one imaging plane (axial). Additionally, this investigation was limited by the reconstruction algorithm, as well as scanners from one vendor. Furthermore, additional sequences should be investigated in future studies with thin-slice imaging, e.g., DWI or DCE imaging.

## 5. Conclusions

This study demonstrates the feasibility of thin-slice T2-weighted TSE imaging of the prostate, including DLR, within a similar acquisition time. The smaller slice thickness and improvement in image quality might lift prostate MRI to the next level.

## Figures and Tables

**Table 1:** MRI acquisition parameters.

	T2_S_	T2_DLR_
TR (ms)	4470	6550
TE (ms)	81	81
Averages	3	2
Voxel size (mm)	0.5 × 0.5 × 3.0	0.5 × 0.5 × 2.0
Field of view (mm)	200	200
Slice thickness (mm)	3	2
Number of slices	30	44
Parallel imaging factor	3	3
Acquisition time (min:sec)	4:12	4:37

**Table 2:** Patients’ characteristics.

Characteristics	Values
Number of patients	N = 30
Age, mean ± standard deviation	68 ± 8 years
Sex	100% male
PSA, median (interquartile range) ^1^	7.5 ng/mL (2.7–12 ng/mL)
*Biopsies*	N = 20
No malignancy	N = 7
*Gleason grading*	
6	N = 1
7a	N = 4
7b	N = 2
8	N = 2
10	N = 3
*Other*	N = 1 (neuroendocrine carcinoma)
*Prostatectomy*	N = 4
T2a	N = 1
T2c	N = 2
T3a	N = 1

^1^ In three cases not available.

**Table 3:** Median (interquartile range) image quality in standard T2-weighted imaging (T2_S_) and deep learning reconstructed thin-slice T2-weighted imaging (T2_DLR_). Likert-scale ranging from 1–4 with 4 being the best rating.

Characteristics	Reader 1			Reader 2		
	T2_S_	T2_DLR_	*p*-Value	T2_S_	T2_DLR_	*p*-Value
Image noise	4 (4–4)	3.5 (3–4)	<0.001	4 (3.75–4)	3.5 (3–4)	0.021
Artifacts	3 (3–3)	4 (4–4)	<0.001	3 (3–4)	4 (4–4)	<0.001
Sharpness	3 (3–3)	4 (4–4)	<0.001	3 (3–3)	4 (4–4)	<0.001
Lesion detectability	3 (3–4)	4 (4–4)	<0.001	3 (3–4)	4 (4–4)	<0.001
Overall image quality	3 (3–3)	4 (4–4)	<0.001	3 (3–3.25)	4 (4–4)	<0.001
Diagnostic confidence	3 (3–4)	4 (4–4)	<0.001	4 (3–4)	4 (4–4)	0.004

**Table 4:** T2 and PI-RADS scoring (29 cases).

T2 and PI-RADS Scoring	Reader 1		Reader 2	
	T2_S_	T2_DLR_	T2_S_	T2_DLR_
*T2 score*				
1	0	0	0	0
2	9	9	9	9
3	5	5	5	5
4	8	8	7	7
5	7	7	8	8
*PI-RADS score*				
1	0	0	0	0
2	9	9	9	9
3	3	3	3	3
4	10	10	9	9
5	7	7	8	8

Lesion size analysis revealed no significant differences between T2_S_ and T2_DLR_ for both readers (Table 5). In three cases both readers suspected extraprostatic growth regardless of the sequence (no histopathological confirmation was available).

**Table 5:** Lesion size measurement in PI-RADS ≥ 3 (20 cases).

Characteristics	Reader 1			Reader 2		
	T2_S_	T2_DLR_	*p*-Value	T2_S_	T2_DLR_	*p*-Value
Lesion size (mm); median (interquartile range)	12.5 (9–19.5)	12.5 (10–19.25)	0.072	13.5 (9–20.25)	12.5 (9.25–19)	0.408

## Data Availability

The datasets used and analyzed during the current study are available from the corresponding author on reasonable request.

## Abbreviations

mpMRI	Multiparametric magnetic resonance imaging;
TSE	Turbo spin echo;
DLR	Deep learning image reconstruction;
GRE	Gradient echo;
T2_S_	Standard T2-weighted TSE imaging;
T2_DLR_	Deep learning reconstructed T2-weighted TSE imaging;
DCE	Dynamic contrast-enhanced;
DWI	Diffusion-weighted imaging;
PI-RADS	Prostate imaging reporting and data system;
PSA	Prostate specific antigen;
IQR	Interquartile range.
